# Mechanistic Insights into 3-Isopropylphenol-Induced Neurotoxicity in Zebrafish: A Network Toxicology and Molecular Docking Approach

**DOI:** 10.3390/toxics13040274

**Published:** 2025-04-03

**Authors:** Jie Gu, Huilin Jin, Jun Hu, Jian Wang, Daqiang Yin

**Affiliations:** 1 Key Laboratory of Yangtze River Water Environment, Ministry of Education, College of Environmental Science and Engineering, Tongji University, Shanghai 200092, China; gujie@nies.org; 2 Nanjing Institute of Environmental Sciences, Ministry of Ecology and Environment, Nanjing 210042, China; 19850866230@163.com (H.J.); hujun@nies.org (J.H.)

**Keywords:** network toxicology, molecular docking, 3-isopropylphenol, neurotoxicity

## Abstract

Endocrine-disrupting chemicals (EDCs) are exogenous substances discharged into the environment through human activities. 3-Isopropylphenol, a typical alkylphenol-based EDC, has been extensively studied due to its broad application and potential ecological impacts. However, the mechanism of its neurotoxicity remains unclear. In this study, the neurotoxic effects of 3-isopropylphenol were examined using the zebrafish model. We predicted its potential toxic mechanisms and action targets using network toxicology and molecular docking and verified them via RT-qPCR. Results showed that 3-isopropylphenol exposure inhibits the cAMP/PKA signaling pathway in zebrafish larvae, promoting apoptosis, impairing neural development, and suppressing locomotor behavior. These findings enhance our understanding of the toxic effects and mechanisms of 3-isopropylphenol on zebrafish larval neural development and aid in evaluating its potential ecological hazards.

## 1. Introduction

Endocrine-disrupting chemicals (EDCs) are exogenous chemical substances released into the environment due to human production and daily activities. They are hydrophobic, lipophilic, highly persistent, capable of long-distance transmission and bioaccumulation, and can interfere with any aspect of hormonal action [1]. This type of pollutant is widely distributed in various environmental compartments such as water, sediment, soil, and the atmosphere, posing a significant potential threat to the ecological environment and human health [2]. Studies have shown that during the prenatal period of organ and nervous system formation and the early postnatal stage, individuals are most vulnerable to the risks posed by EDCs [3].

Among them, 3-isopropylphenol, as a typical alkylphenol-based endocrine-disrupting chemical, is commonly found in products like surfactants, industrial additives, and personal care products [2]. Its extensive application and potential ecological risks have drawn widespread attention. Studies have shown significant variations in phenolic compound concentrations across different river basins. For example, in the Southeast Rivers, the Yellow River, the Pearl River, and the Huai River, phenolic compound concentrations range from 12~44,650 ng/L, 2.4~27,490 ng/L, 9.7~5046.9 ng/L, and 4.4~3010.9 ng/L, respectively. Notably, the average concentration of certain phenolic compounds (e.g., nonylphenol) far surpasses the European Union standard (330 ng/L), highlighting a severe environmental pollution issue [1].

When 3-isopropylphenol enters environmental media such as water and soil, it can persist in the environment and may accumulate in organisms through the food chain. Such accumulation may not only cause negative effects on organisms, such as endocrine disrupting activity, hepatotoxicity, genotoxicity, etc., but may also pose a potential threat to human health [4]. For instance, studies have detected 3-isopropylphenol concentrations as high as 8 μg/kg in contaminated fish (e.g., snakehead, pike, and yellow perch) from the Wisconsin River [5], underscoring its widespread environmental distribution and bioaccumulation.

To date, research on the environmental toxicity of 3-isopropylphenol has been limited, with its underlying mechanisms still unclear. Traditional toxicological studies often focus on isolated action patterns of single or multiple targets, falling short in comprehensively revealing the systematic impacts and related toxic pathways of emerging pollutants that trigger complex toxic effects by interfering with various biological molecular networks. Consequently, a comprehensive evaluation of their toxic mechanisms remains elusive. The integration of network toxicology and molecular docking technology has emerged as a promising solution to this challenge [6]. Network toxicology, an interdisciplinary field encompassing bioinformatics, big data analysis, and genomics [7,8], employs a network-based approach to elucidate how interactions among different molecules in biological systems lead to toxicity, shifting the paradigm from “one target, one drug” to “multi-target, one drug” [9]. Molecular docking, a computer-based structure-driven method, predicts ligand–target interactions at the molecular level and depicts structure–activity relationships without prior knowledge of the chemical structures of other target modulators [10]. In this study, we combined network toxicology and molecular docking technology to predict the toxicity of 3-isopropylphenol, providing a theoretical basis for investigating its neurotoxicity to zebrafish larvae and the underlying mechanisms.

Zebrafish, widely used in environmental pollutant studies [11], has neurological development remarkably similar to mammals [12]. Due to their small size, low cost, and rapid development [13,14], they are extensively used in toxicity evaluations. Moreover, the optical transparency of zebrafish embryos enables researchers to observe individual cells during development, facilitating in vivo imaging and dynamic tracking within the whole organism. With a relatively short generation cycle of 3–4 months, zebrafish are also suitable for genetic research. Based on these characteristics, zebrafish are highly suitable for transgenic technology to express green fluorescent protein (GFP) or other fluorescent proteins [15,16]. Transgenic lines like *Tg* (*huc:eGFP*) and *Tg* (*hb9:eGFP*), which allow direct observation of neuron development and motor neuron axon growth [17], have been widely applied in toxicological research [18].

The primary aim of this study is to explore the neurotoxic effects of 3-isopropylphenol through the zebrafish model. Furthermore, techniques like network toxicology, molecular docking, and RT-qPCR are employed to predict and validate the toxic mechanisms and action targets of 3-isopropylphenol. At present, as a substance with potential endocrine-disrupting effects, studying 3-isopropylphenol not only helps us better understand its mechanism of action but also provides crucial evidence for the safe use of related products and environmental risk assessment, thus maintaining ecological balance and human health.

## 2. Materials and Methods

### 2.1. Chemicals and Reagents

3-Isopropylphenol (CAS: 618-45-1, >98% purity) was purchased from Shanghai Aladdin Biochemical Technology Co., Ltd. (Shanghai, China). Dimethyl sulfoxide (DMSO) (CAS: 67-68-5) was purchased from Beijing Solarbio Science & Technology Co., Ltd. (Beijing, China). All chemical reagents and solvents employed in the investigation were of analytical grade. The TRIzol reagent was purchased from Takara, Dalian, China. The SYBR Green kit was purchased from Nanjing Vazyme Biotech Co., Ltd. (Nanjing, China). The gene primers were synthesized by Sangon Biotech (Shanghai) Co., Ltd. (Shanghai, China).

### 2.2. Breed and Rearing of Zebrafish

Wild-type AB strain zebrafish and transgenic strains *Tg* (*huc:eGFP*) and *Tg* (*hb9:eGFP*) were obtained from the Institute of Hydrobiology, Chinese Academy of Sciences (IHB, Wuhan, China). The wild-type AB strain was used to study some basic indicators, such as fetal movement, body length, heart rate, and other indicators; *Tg* (*huc:eGFP*) zebrafish was used to study the influence of central nervous system neuron development; and *Tg* (*hb9:eGFP*) was used to study the influence of motor neuron axon growth. Adult zebrafish were kept in a recirculating water system with a water temperature of 28 ± 0.5 °C and a photoperiod of 14 h light/10 h dark and were fed twice daily with live Artemia. To obtain fertilized eggs, male and female zebrafish were placed in a breeding tank at a ratio of 1:2 the day before spawning. Early the next morning, the partition boards were removed to allow the zebrafish to spawn naturally under light conditions. Then, healthy embryos were selected under a microscope for the subsequent exposure experiment. All animal experiments were conducted in accordance with the guidelines for the care and use of laboratory animals of the Nanjing Institute of Environmental Sciences (IACUC—20240912).

### 2.3. Construction of Targets for 3-Isopropylphenol and Nerve Injury

The construction of core targets for 3-isopropylphenol-induced nerve injury is mainly achieved through the intersection of 3-isopropylphenol targets and nerve injury targets. The specific steps to construct the target of 3-isopropylphenol were as follows: First, the keyword “3-isopropylphenol” was entered in the PubChem database (https://pubchem.ncbi.nlm.nih.gov (accessed on 23 December 2024)) and the best match was found, ensuring that the name and molecular formula were accurate. Next, the SDF file was downloaded in the 2D Structure section of the retrieval page. Finally, we uploaded the result to the Swiss Target Prediction database (http://www.swisstargetprediction.ch/ (accessed on 23 December 2024)); the prediction condition probability was set to be greater than 0, and it was submitted to download the prediction results. The construction steps of nerve injury targets were as follows: The keyword “nerve injury” was entered in the GeneCards database (http://www.genecards.org/ (accessed on 23 December 2024)), and the results would be all the targets of nerve injury. After obtaining the 3-isopropyl phenol and nerve injury targets, respectively, they were uploaded to the Venn website (http://bioinformatics.psb.ugent.be/webtools/Venn/ (accessed on 23 December 2024)) to obtain their intersection.

### 2.4. Construction of the PPI Network

The construction of the protein–protein interaction (PPI) network mainly utilizes the STRING database (https://cn.string-db.org/ (accessed on 25 December 2024)). The overlapping targets obtained from the Venn diagram of 3-isopropylphenol and nerve injury were uploaded to the STRING database. In order to ensure that we can accurately analyze the active target proteins corresponding to the target genes, the specific parameters were set as follows: the species limit was “Homo sapiens”, “Minimum required interaction score” was set as “High Confidence > 0.9”, and the “FDR Stringency Value” was selected as “High”, for analysis. The network structure and key nodes were analyzed and optimized using Cytoscape 3.10.3 software. The requirement for selecting core targets is that the degree value is greater than or equal to twice the median.

### 2.5. GO and KEGG Pathway Analysis

The intersecting targets obtained from the Venn diagram were uploaded to the DAVID database (https://david.ncifcrf.gov/ (accessed on 10 January 2025)), where the species Homo sapiens was selected to acquire Gene Ontology (GO) and Kyoto Encyclopedia of Genes and Genomes (KEGG) pathway analysis data associated with 3-isopropylphenol-induced neurotoxicity. Subsequent graphical representations were generated for the KEGG pathway enrichment results (top 20 entries, ordered by ascending *p*-value) and GO enrichment outcomes (top ten entries per subclass, encompassing biological, cellular, and molecular categories).

### 2.6. Molecular Docking

The software mainly used for molecular docking is Autodock Tools 1.5.6 and Pymol 2.3.4. Firstly, the ligand needs to be prepared. The ligand can be queried in the PDB database (https://www.rcsb.org). The resolution should be selected to be above 2.0, and the species is selected as human. After downloading the SDF structure file of the ligand from PubChem, it is converted into a PDB format file. Then, the ligand was hydrogenated, and water molecules were removed using the Autodock Tools 1.5.6 software. The processed ligand was saved as a PDBQT file for subsequent docking simulations. Secondly, water molecules and ligands were removed from the protein using Pymol 2.3.4, and it was saved in the PDB format. Next, the pdbqt format of the receptor was obtained and saved using the Autodock Tools 1.5.6 software. After the size of the Gridbox is finally determined, the Autodock Tools 1.5.6 software was run using the CMD command characters for molecular docking. For the visualization of the molecular docking results, Discovery Studio 2024 and Pymol 2.3.4, can be applied for presentation.

### 2.7. 3-Isopropylphenol Exposure and General Developmental Toxicity Testing

Ten wild-type zebrafish embryos were placed in each well of a 6-well plate with 5 mL of aerated water, with three replicates per concentration. The exposure concentrations of 3-isopropylphenol were 100, 200, 400, 800, 1600, and 3200 μg/L until 96 h post-fertilization (hpf). The solution was renewed daily, and embryos were observed three times daily. Dead embryos were recorded and removed. The median lethal concentration (LC_50_) was calculated based on the data. According to the acute toxicity test results and environmental concentration [5], the concentrations of 3-isopropylphenol were set at 0, 1, 10, and 100 μg/L. Thirty transgenic zebrafish embryos were placed in each Petri dish with 20 mL of the exposure solution, with three replicates per concentration for microscopic observation. During the experiment, a fluorescence stereomicroscope was used to record mortality and hatching rate every 24 h. Whitened and coagulated embryos were removed to prevent contamination. At 24 hpf, photographs were taken to measure the yolk sac area and embryonic movement of zebrafish embryos. At 72 hpf, photographs were taken to count the heart rate and measure the body length of zebrafish. At 144 hpf, photographs were taken to measure the body length and the area around the eyes of zebrafish (n = 12).

### 2.8. Behavioral Testing of Zebrafish Larvae

The methods for larval behavioral tests are based on previously published methods [17]. Wild-type zebrafish embryos were exposed to 3-isopropylphenol at a concentration of 0, 10, 100, and 1000 μg/L. The locomotor behavior of 6-day post-fertilization (dpf) zebrafish larvae was tested by using the stimulation of an alternating light–dark–light cycle. Twelve zebrafish larvae from each treatment group were taken and transferred to 24-well plates (one larva per plate) to which 3 mL of test water was added. The larvae were acclimatized at 28 ± 0.5 °C for 10 min, and then their locomotor activity was recorded. Test conditions consisted of alternating light and dark stimuli (10 min light: 10 min dark, for a total of 40 min). Locomotor behavior was recorded by DanioVision XT 16 software (Noldus, Wageningen, The Netherlands), and zebrafish dull time, activity, acceleration, and frequency of activity under light–dark conditions were calculated and statistically analyzed using the Animal Trajectory Tracking System XT15 software. Each experiment was repeated three times (n = 12).

### 2.9. Neurodevelopmental Toxicity Studies in Zebrafish Larvae

Given the observed impact of 3-isopropylphenol on zebrafish locomotion, transgenic zebrafish strains *Tg* (*huc:eGFP*) and *Tg* (*hb9:eGFP*) were exposed to varying concentrations of 3-isopropylphenol to assess its effects on neuronal development. Specimens (n = 12) were to be randomly selected from each exposure group and fixed with 4% paraformaldehyde for 5 min. All experimental procedures were carried out in triplicate. Using a fluorescence stereomicroscope (Nikon SMZ25, Tokyo, Japan), after exposure to 3-isopropylphenol until 72 hpf and 144 hpf, statistically analyze the fluorescence expression intensity of transgenic *Tg* (*huc:eGFP*) and the length of neural axons of transgenic *Tg* (*hb9:eGFP*). The NIS-Elements D 5.20.00 software was used to quantitatively analyze the fluorescence intensity of the fluorescent proteins and the axon lengths in zebrafish larvae.

### 2.10. Real-Time Fluorescence Quantitative PCR

Fifty zebrafish embryos were placed in each Petri dish containing 25 mL of the exposure solution. Each group was exposed to 3-isopropylphenol until 144 hpf, and then the embryos were collected into 1.5 mL eppendorf tubes. Three replicates were set for each concentration. Finally, the total RNA was extracted by using Trizol reagent (Takara, Dalian, China). The ratio of 260/280 for a pure RNA sample is between 1.8 and 2.2. cDNA was synthesized using the PrimeScript^®^ RT kit. Gene expression analysis was performed on a Bio-Rad CFX Connect real-time system (Bio-Rad, Hercules, CA, USA) using the SYBR Green detection method (Vazyme Biotech Co., Ltd., Nanjing, China). The expression levels of genes related to neural development (*elav3*, *gfap*, *syn2a*, and *gap43*), genes related to the cAMP/PKA pathway (*drd1*, *drd2a*, *cAMP*, *PKA*, *CREB*, *gnb1b*, and *gng2*), and genes related to apoptosis (*bad* and *bcl-2*) were quantitatively analyzed using the primers synthesized by Sangon Biotech Co., Ltd. (Shanghai, China). The specific details are shown in Table S1. *β*-actin was used as an internal reference, and the relative RNA levels were calculated using the 2^−ΔΔCt^ method.

### 2.11. Statistical Analysis

Statistical analysis was performed using GraphPad Prism 8 (GraphPad Software, San Diego, CA, USA). The Kolmogorov–Smirnov test was employed to assess the normality of the data distribution. One-way analysis of variance (ANOVA) was used, followed by Tukey’s test to evaluate the significant differences among groups. A *p*-value less than 0.05 was considered statistically significant. The significant differences between the exposure groups and the control group were denoted as follows: *p* < 0.001 (***), *p* < 0.01 (**), and *p* < 0.05 (*).

## 3. Results

### 3.1. Prediction of Neurotoxicity Induced by 3-Isopropylphenol

**Figure 1:**
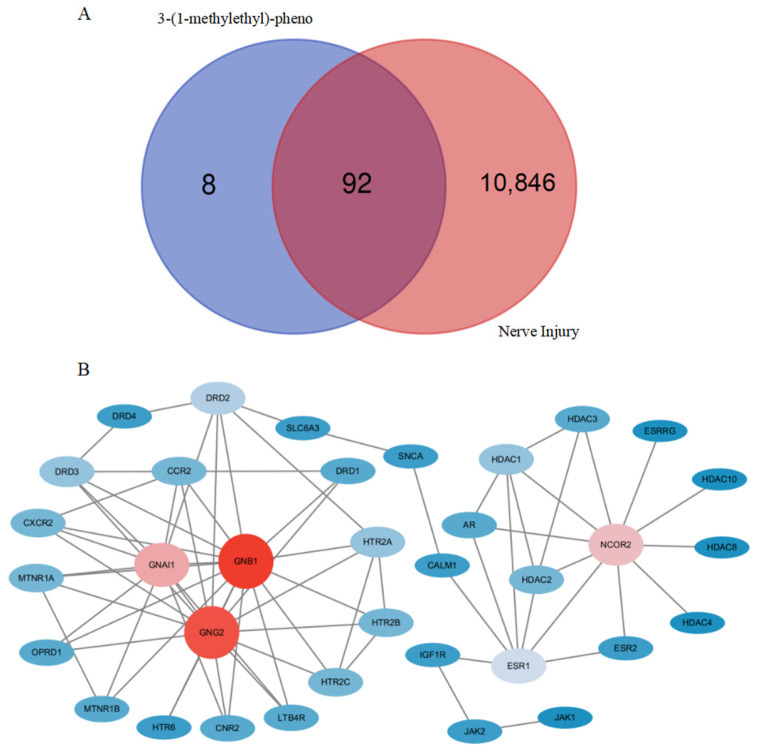
Prediction of the toxicity of 3-isopropylphenol based on network toxicology. (**A**) Venn diagram of the toxicity targets of 3-isopropylphenol and the targets related to nerve injury. (**B**) The PPI network of the potential targets, the darker the color of a node, the higher its degree centrality.

In this study, using the SwissTarget Prediction and Genecards databases, we screened out 100 potential 3-isopropylphenol targets and 10,938 nerve injury-related targets. After integrating these target sets and removing duplications, we obtained 92 intersecting targets that may underlie 3-isopropylphenol-induced neurotoxicity (Figure 1A).

### 3.2. The Main Interaction Network and Core Genes of Potential Targets

We constructed a PPI using the STRING database. The network consisted of 92 nodes and 126 edges, with an average node degree value of 2.74. The topological properties of the network nodes, including degree and betweenness centrality, were analyzed using the Cytoscape 3.10.3 software. Meanwhile, a visualized and optimized main protein–protein interaction network diagram was generated (Figure 1B). Through network analysis, a group of 35 core targets for the neurotoxicity induced by 3-isopropylphenol was determined, and the results are shown in Table S2. It is worth noting that the top three targets ranked according to the degree value are G protein subunit beta 1 (GNB1), G protein subunit gamma 2 (GNG2), and G protein subunit alpha I1 (GNAI1), and the core targets are GNB1 and GNG2. The proteins encoded by these genes can induce neurological diseases such as intellectual developmental disorder, global developmental delay, neurodevelopmental disorder with hypotonia, speech disorder, and behavioral disorder.

### 3.3. Target Functional Analysis and Pathway Enrichment Analysis

**Figure 2:**
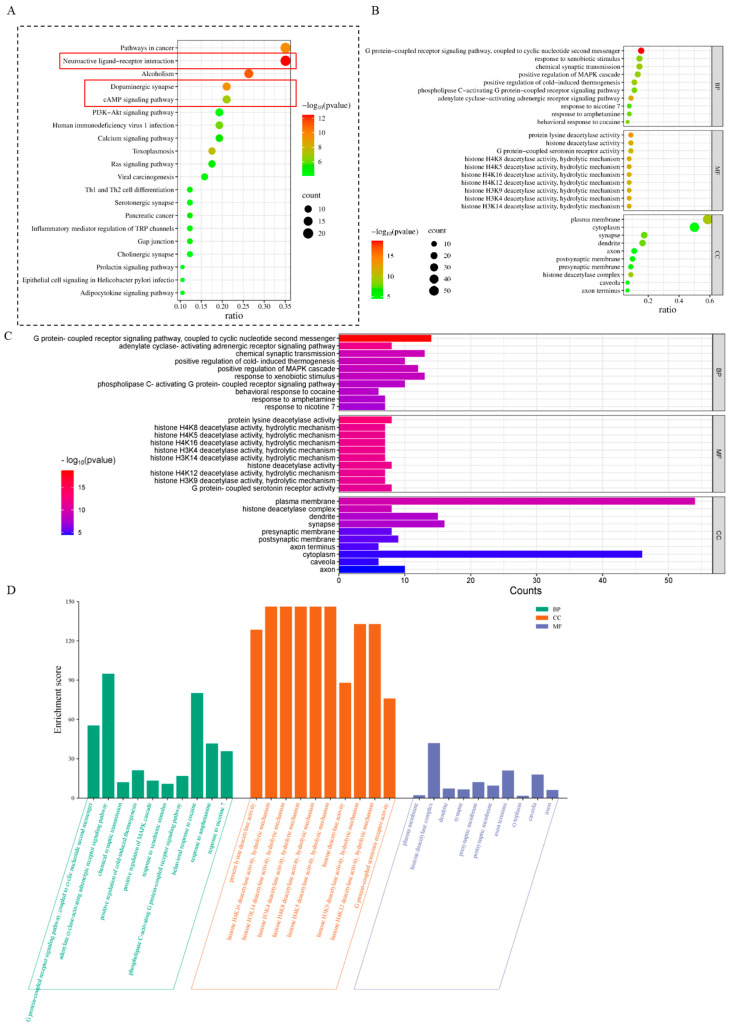
Target functional analysis and pathway enrichment analysis of 3-isopropylphenol. (**A**) KEGG enrichment analysis of the potential targets (the main signalling pathways are highlighted in the red box.) (**B**–**D**) GO enrichment analysis (biological process (BP), cellular component (CC), and molecular function (MF)) of the potential targets. (**C**) The number of genes of the potential targets (biological process (BP), cellular component (CC), and molecular function (MF)).

To explore the potential toxic pathways of 3-isopropylphenol, we used the DAVID database to conduct a KEGG analysis on these 92 potential targets to determine the specific signaling pathways they are involved in. We generated a significant statistical bubble plot to visually display the top 20 KEGG signaling pathways according to the magnitude of the *p*-value (Figure 2A). Moreover, we performed a GO analysis on the 92 potential targets, limiting the species to Homo sapiens. The analysis yielded a total of 457 statistically significant GO terms, among which there were 278 in biological process (BP), 57 in cellular component (CC), and 122 in molecular function (MF). The GO terms were ranked according to the *p*-value. Among the biological process, cellular component, and molecular function, the top 10 terms with the lowest *p*-value were selected and intuitively presented in the enrichment analysis graphs (Figure 2B,D). The number of genes in the GO terms is shown in Figure 2C.

In the enrichment of KEGG signaling pathways, various signal-related pathways emerged significantly, including the neuroactive ligand–receptor interaction, dopaminergic synapse, and cyclic adenosine monophosphate (cAMP) signaling pathway. Moreover, in the GO terms, the G protein-coupled receptor signaling pathway coupled to cyclic nucleotide second messengers also emerged significantly. These findings are consistent with the types of neurodevelopmental damage.

### 3.4. Molecular Docking of 3-Isopropylphenol and the Core Target Proteins Related to Nerve Injury

**Figure 3:**
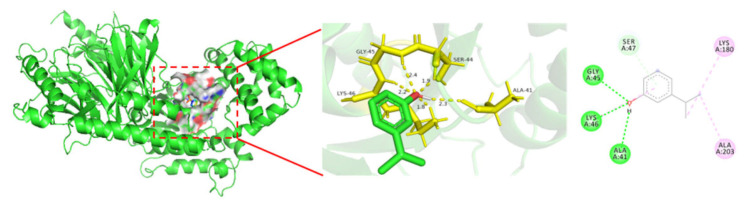
Molecular docking results of the 6C2Y protein with the lowest binding energy to 3-isopropylphenol.

To explore the interactions between 3-isopropylphenol and the two core target genes (GNB1 and GNG2), a molecular docking analysis was carried out. Firstly, we obtained the three-dimensional structure of the small molecule drug ligand of 3-isopropylphenol from the PubChem database and optimized it. In addition, we retrieved the crystal structure of the target protein receptor (ID: 6C2Y) from the Protein Data Bank database. The AutoDockTools 1.5.6 software (developed by the Scripps Research Institute in the United States) was used to perform molecular docking between the small molecule ligand and the protein receptor. The results showed that the binding energy was −5.1 kcal/mol, indicating a strong affinity between 3-isopropylphenol and the targets, which highlights their important roles in the neurotoxicity induced by 3-isopropylphenol and the molecular mechanism. Using Pymol 2.3.4 and Discovery Studio 2024 software, the visual image of the lowest binding energy between the targets and the compound was drawn (Figure 3).

### 3.5. Effects of Exposure to 3-Isopropylphenol on the Early Development of Zebrafish Larvae

**Figure 4:**
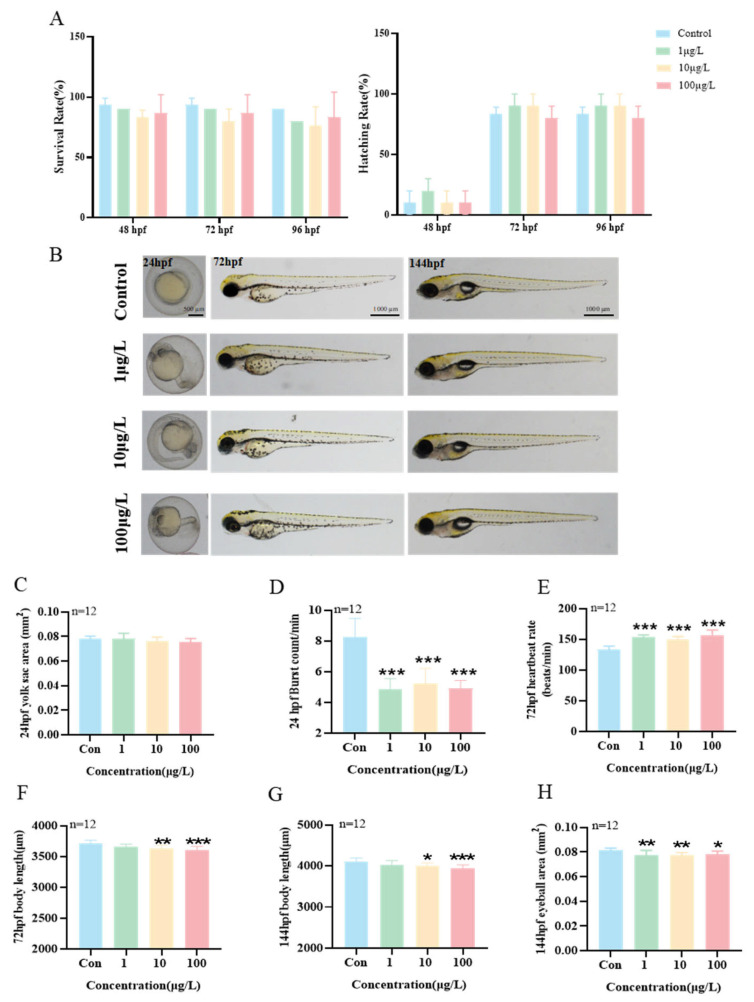
Effects of exposure to 3-isopropylphenol on the early development of zebrafish larvae. (**A**) Survival rate and hatching rate of zebrafish larvae after exposure until 96 hpf. (**B**) Typical images of the early development of zebrafish larvae. (**C**) Yolk sac area and (**D**) embryonic movement of zebrafish embryos after exposure until 24 hpf. (**E**) Heart rate and (**F**) body length of zebrafish larvae after exposure until 72 hpf. (**G**) Body length and (**H**) eye circumference area of zebrafish larvae after exposure until 144 hpf. The data are presented as the mean ± standard deviation of three independent experiments (n = 12). *p* values: *p* < 0.001 (***), *p* < 0.01 (**), and *p* < 0.05 (*).

3-Isopropylphenol has an adverse impact on the early growth and development of zebrafish larvae. First, the 96 hpf median lethal concentration (LC_50_) of zebrafish was calculated to be 1 mg/L. Then, zebrafish embryos were exposed to different concentrations of 3-isopropylphenol (1, 10, and 100 μg/L) and observed from 4 hpf to 144 hpf (Figure 4B). The results showed that, compared with the control group, there was no significant difference in the survival rate and hatching rate of zebrafish embryos exposed to 3-isopropylphenol at ≤100 μg/L (Figure 4A). At 24 hpf, exposure to different concentrations of 3-isopropylphenol did not affect the yolk sac area of zebrafish embryos (Figure 4C), but starting from 1 μg/L, the number of spontaneous movements of embryos per minute decreased significantly (Figure 4D, *p* < 0.001). When exposed to 72 hpf and 144 hpf, 3-isopropylphenol at concentrations of 10 μg/L and higher significantly inhibited the body length of zebrafish larvae (Figure 4F,G), and at 144 hpf, concentrations of 1 μg/L and higher significantly reduced the area of the eyes (Figure 4H, *p* < 0.01). Interestingly, compared with the control group, the heart rate of larvae exposed to 1 μg/L 3-isopropylphenol at 72 hpf increased significantly (Figure 4E, *p* < 0.001). The above results show that exposure to 3-isopropylphenol can impact the early growth and development of zebrafish larvae in multiple aspects, spanning from zebrafish embryo hatching to larval morphological development.

### 3.6. The Effects of 3-Isopropylphenol on the Motor Behavior of Zebrafish Larvae

**Figure 5:**
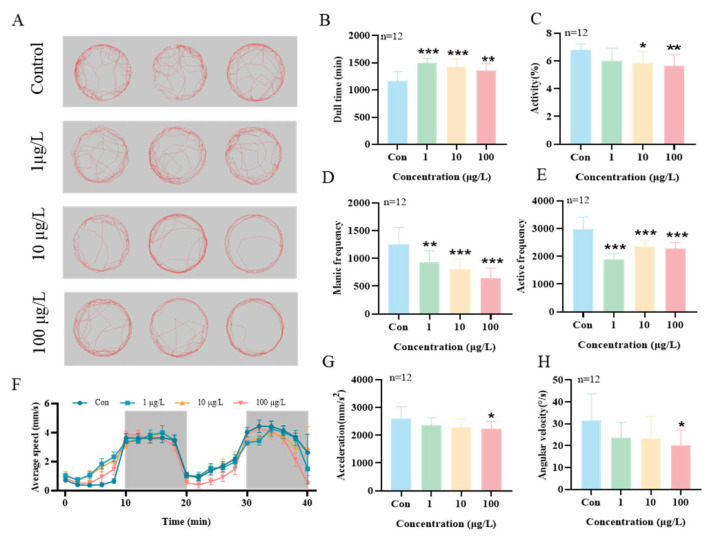
Effects of exposure to 3-isopropylphenol on the locomotor behavior of zebrafish larvae. (**A**) Trajectories of locomotor behavior, (**B**) immobility time, (**C**) activity level, (**D**) frequency of manic behavior, (**E**) frequency of active behavior, (**F**) average locomotion speed, (**G**) acceleration, and (**H**) angular velocity of zebrafish larvae after exposure until 144 hpf. The data are presented as the mean ± standard deviation of three independent experiments (n = 12). *p* values: *p* < 0.001 (***), *p* < 0.01 (**), and *p* < 0.05 (*).

Zebrafish embryos were continuously exposed to 3-isopropylphenol until 144 hpf, and the behavioral indices of zebrafish larvae were observed. Figure 5A shows the movement trajectories of zebrafish larvae at 144 hpf under light and dark stimuli. The results showed that exposure to 3-isopropylphenol led to a decrease in the movement speed under light and dark alternating stimuli (Figure 5E), and the stagnation time significantly increased starting from 1 μg/L (Figure 5B, *p* < 0.001). When the exposure concentrations were 1 μg/L, 10 μg/L, and 100 μg/L, the stagnation time increased by 27.8%, 21.5%, and 16.5%, respectively. The activity of zebrafish larvae significantly decreased starting from 10 μg/L (Figure 5C, *p* < 0.05), and when the exposure concentrations were 10 μg/L and 100 μg/L, the activity decreased by 13.8% and 16.9%, respectively. The frequencies of mania and activity of zebrafish larvae significantly decreased as the concentration increased (Figure 5D,E). When the exposure concentration was 100 μg/L, the acceleration and angular velocity significantly decreased compared with the control group (Figure 5G,H, *p* < 0.05). These results indicate that exposure to 3-isopropylphenol inhibits the motor activity of zebrafish larvae, and it is speculated that it may affect the development of the nervous system.

### 3.7. The Effects of 3-Isopropylphenol on the Nervous System of Zebrafish Larvae

**Figure 6:**
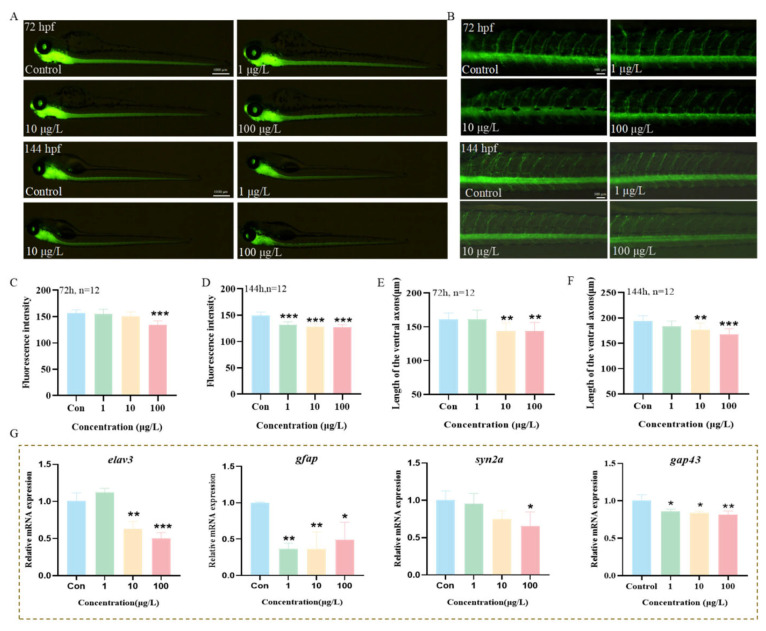
Effects of exposure to 3-isopropylphenol on the neural development of zebrafish larvae. Typical images of transgenic zebrafish strains (**A**) *Tg* (*huc:eGFP*) and (**B**) *Tg* (*hb9:eGFP*) after exposure until 72 hpf and 144 hpf. Fluorescence intensity of zebrafish larvae at (**C**) 72 hpf and (**D**) 144 hpf and the length of neural axons at (**E**) 72 hpf and (**F**) 144 hpf. (**G**) Genes related to neural development. The data are presented as the mean ± standard deviation of three independent experiments (n = 12). *p* values: *p* < 0.001 (***), *p* < 0.01 (**), and *p* < 0.05 (*).

Motor disorders are closely related to the nervous system [19]. To further investigate the effects of 3-isopropylphenol on the nervous system of zebrafish larvae, transgenic zebrafish strains *Tg* (*huc:eGFP*) with fluorescent labeling of the central nervous system and *Tg* (*hb9:eGFP*) with labeling of motor neurons were exposed to 3-isopropylphenol until 144 hpf. A fluorescent stereomicroscope was used to observe and quantify the effects of 3-isopropylphenol on the nervous system at 72 hpf and 144 hpf (Figure 6A,B). The results showed that when exposed to 72 hpf and 144 hpf, the green fluorescence intensity in the brain and spinal cord of *Tg* (*huc:eGFP*) zebrafish began to decrease starting from 100 μg/L and 1 μg/L, respectively (Figure 6C,D, *p* < 0.001). In addition, in *Tg* (*hb9:eGFP*) zebrafish, when the concentration of 3-isopropylphenol was ≥10 μg/L, the axon length of motor neurons was significantly shortened (Figure 6E,F, *p* < 0.01). The genes related to neural development (*elav3*, *gfap*, *syn2a*, and *gap43*) were significantly inhibited (Figure 6G), further verifying that 3-isopropylphenol has an adverse effect on the neural development of zebrafish larvae.

### 3.8. 3-Isopropylphenol Affects the cAMP/PKA Signaling Pathway in the Body of Zebrafish Larvae

**Figure 7:**
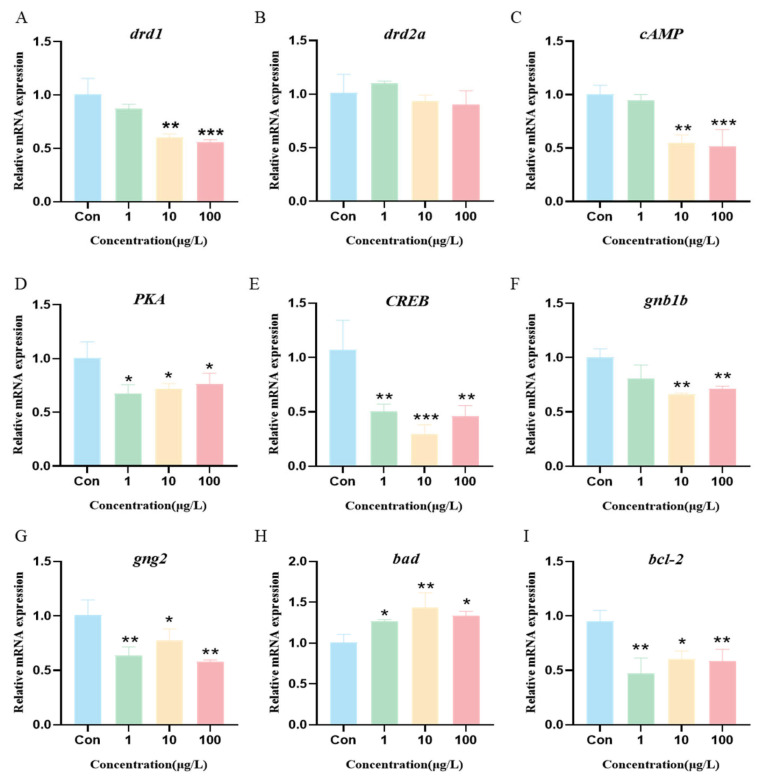
Expression of genes related to cAMP/PKA signaling pathway and apoptosis. (**A**–**G**) Genes related to the cAMP/PKA pathway. (**H**,**I**) Genes related to apoptosis. *p* values: *p* < 0.001 (***), *p* < 0.01 (**), and *p* < 0.05 (*).

To further explore how 3-isopropylphenol affects zebrafish larval neural development, we exposed zebrafish embryos to 3-isopropylphenol until 144 hpf. Then, we extracted RNA and conducted a quantitative analysis of gene expression levels in the cAMP/PKA signaling pathway. The results showed that when the exposure concentration was 10 μg/L, the expression of the dopamine receptor *drd1* gene was significantly inhibited (Figure 7A, *p* < 0.01), while there was no significant difference in the expression of *drd2a* (Figure 7B). At the same time, compared with the control group, the expression levels of *cAMP*, *PKA*, and *CREB* genes decreased significantly (Figure 7C–E). Among them, the expression levels of the core target genes *gnb1b* and *gng2* also decreased accordingly (Figure 7F,G). These results indicate that 3-isopropylphenol inhibits the transduction of the cAMP/PKA signal in zebrafish larvae. There were significant differences in the expression of the *bad* and *bcl-2* starting from an exposure concentration of 1 μg/L (Figure 7H,I), suggesting that 3-isopropylphenol induces apoptosis of neural cells in zebrafish larvae.

## 4. Discussion

Network toxicology, as a novel approach, is of great significance in analyzing the interactions and regulations of toxic substances in biological systems and elucidating the mechanisms of toxic effects [20]. In this study, 3-isopropylphenol, a typical endocrine disruptor of great environmental significance, is selected for research. However, current studies on its environmental behavior and ecological toxicity are still relatively limited. Only a small number of existing studies have explored the toxicological effects of this substance. In this study, we first applied a series of network toxicity prediction tools. Then, using the Swiss Target Prediction and GeneCards databases, we screened for targets related to 3-isopropylphenol-induced nerve injury. Based on the STRING platform and Cytoscape 3.10.3 software, we constructed an interaction network of potential targets. From this network, GNB1 and GNG2 were identified as core targets for 3-isopropylphenol-induced neurotoxicity in zebrafish larvae. Additionally, experiments and RT-qPCR technology were used to verify the toxicity and mechanism, providing a complete chain of evidence.

GNB1 (G protein subunit β1) is a gene encoding a subunit of the guanine nucleotide-binding protein [21]. Mutations in this gene can lead to neurodevelopmental disorders in the head region, including global developmental delay and epilepsy, resulting in GNB1 encephalopathy [22]. Previous studies have shown that GNB1 encephalopathy can cause neurodevelopmental disorders by altering the signaling of potassium (GIRK) channels [23,24]; moreover, as a member of the Gβ subunits, GNB1 can form a dimer with the Gγ subunit. After the activation of G protein-coupled receptors (GPCRs), this dimer can dissociate from the Gα subunit, thereby regulating downstream effector molecules, including adenylate cyclase [25,26]. The other core target, GNG2, is one of the genes encoding the γ subunit of the guanine nucleotide-binding protein. After binding to the Gβ subunit, it may participate in the transmembrane signaling mechanism by regulating the activity of the Gα subunit and affect a variety of cellular physiological processes, including cell proliferation, differentiation, and metabolism [27,28]. Studies have found that GNB1 and GNG2 can indirectly affect the cAMP-PKA pathway, which is consistent with our pathway prediction results. The core targets of the neurotoxicity induced by 3-isopropylphenol are mainly enriched in the neuroactive ligand–receptor interaction, dopaminergic synapse, and cAMP signaling pathway. Previous studies have shown that emerging pollutants can cause neurotoxicity in organisms through these pathways and signaling pathways [29,30,31].

Molecular docking serves as an invaluable tool in exploring and understanding the interactions between chemical substances and their respective biological receptor targets. It not only has the capability to precisely simulate the binding sites and specific types of interactions between a compound and a receptor but can also evaluate the stability of the resulting binding complex. Through these insights, it offers crucial information that paves the way for uncovering the toxic mechanisms of the compound within the target organism [32]. This technology has been widely used to predict and analyze the potential mechanisms of toxicity induced by endocrine disruptors [33,34,35]. The results of this study indicate that the binding energy between 3-isopropylphenol and the proteins of GNB1 and GNG2 is −5.13 kJ/mol, forming a relatively stable complex, which demonstrates that there is an interaction of a certain intensity. After binding, a neuroeffect is produced, reasonably verifying our previous predictions. Network toxicology and molecular docking techniques hold significant positions in toxicological research and drug development. Nevertheless, they are confronted with certain issues, such as data dependence, limitations in mechanism inference, and computational resource constraints. Thus, it is essential to integrate experimental verification to enhance the reliability of the results.

Zebrafish have the characteristics of a unique and moderately high-throughput system, low cost, and a large number of offspring in reproduction. By taking advantage of these features, the toxicity of compounds can be rapidly evaluated, and specific molecular mechanisms can be studied [36]. In this study, it was found that the growth and development of zebrafish embryos were significantly inhibited when they were exposed to 3-isopropylphenol. Previous studies have shown that triphenyl phosphate (TPhP) can affect zebrafish by inhibiting their body length [37], thus having an impact on their growth and development. This is consistent with the results of our experiment. When exposed to 3-isopropylphenol until 144 hpf, the area of the eyes began to decrease significantly starting from an exposure concentration of 1 μg/L. The above research results show that exposure to 3-isopropylphenol has an adverse effect on the early growth and development of zebrafish.

The locomotor behavior of zebrafish, a critical indicator of neural development, has found extensive application in assessing the neurotoxicity of diverse pollutants. Given that the movement of zebrafish larvae is regulated by spinal cord neurons, the locomotor behavior index has emerged as a significant basis for evaluating the neurotoxicity resulting from exposure to environmental pollutants [38]. The results of this study show that the motor behavior of larval zebrafish exposed to 3-isopropylphenol will be significantly inhibited. Previous studies have found that tributyltin (TBT) can affect the locomotor behavior of zebrafish larvae [39], which is consistent with the results of our experiment, indicating that 3-isopropylphenol inhibits the locomotor behavior of zebrafish larvae. Based on the results of the early development and locomotor behavior of zebrafish, it is reasonably speculated that 3-isopropylphenol may have an impact on the nervous system of zebrafish larvae.

The neuroanatomical structure and physiological characteristics of zebrafish are closely linked to their behaviors, showing a high degree of consistency with research findings in mammals. This makes zebrafish a dependable and highly relevant experimental model for studying nervous system diseases [40]. The use of transgenics in fish is a relatively new advancement, which promotes the understanding of genetic mechanisms and developmental processes and provides a more advanced and comprehensive system for evaluating the health impacts of chemicals. Transgenic zebrafish have been used to study the development and regeneration of the nervous system [41]. Green fluorescent protein (EGFP) has been modified through genetic engineering techniques [42], and GFP has been integrated into the promoter of the *elavl3* gene (huc), enabling it to encode a neuron-specific RNA-binding protein [43]. The expression of GFP is driven by the key genes huc and hb9 for the development of the central nervous system and motor nervous system, respectively [44]. We used two transgenic zebrafish lines, *Tg* (*huc:eGFP*) and *Tg* (*hb9:eGFP*), to evaluate the effects of 3-isopropylphenol on the neural development of zebrafish larvae. The results showed that when exposed to 3-isopropylphenol until 72 hpf and 144 hpf, the fluorescence intensity was weakened and the length of the nerve axons was significantly inhibited. Previous studies used *Tg* (*huc:eGFP*) to study the neurotoxicity of six phthalates on zebrafish embryos [45], and the results were consistent with those of our experiment, that is, it slowed down the development of neurons in the central nervous system. In addition, the *syn2a* gene is a gene closely related to the neural development and synaptic function of zebrafish, and gene mutations may also affect the behavior of zebrafish [46]. *Gap43* plays an important role in the differentiation of bone marrow mesenchymal stem cells into ganglion-like cells, guiding axonal growth and regulating synaptic formation [47]. *Gfap* is mainly present in astrocytes, which are the most abundant subtype of glial cells in the brain and spinal cord and play a variety of physiological roles in nervous system diseases [48]. The significant suppression of the expression of genes related to neural development further verifies that the inhibition of the locomotor behavior of zebrafish larvae by exposure to 3-isopropylphenol is positively correlated with the damage to the central nervous system and motor nerves.

The results of pathway enrichment analysis showed that the core targets of the neurotoxicity induced by 3-isopropylphenol are mainly enriched in the neuroactive ligand–receptor interaction, dopaminergic synapse, and cAMP signaling pathway. Dopamine D1 receptor (DRD1) is a transmembrane protein belonging to the GPCR family. It is closely related to functions such as movement and cognition [49]. As is known to all, when dopamine binds to DRD1, it will activate the coupled Gs protein. The Gs protein will further activate adenylate cyclase (AC), causing adenosine triphosphate (ATP) in the cell to be converted into cAMP, thus activating the cAMP/PKA signaling pathway [50]. The results of this study showed that, compared with the control group, when the exposure concentration of 3-isopropylphenol reached 10 μg/L, the expression of the *drd1* gene in zebrafish larvae was significantly inhibited. This indicates that dopamine was unable to effectively activate DRD1, resulting in a decrease in the ability to activate Gs protein and AC. As a result, the production of cAMP was reduced and the activity of PKA was decreased, causing the relevant signaling pathway to be blocked. Cyclic adenosine monophosphate response element-binding protein (CREB) is an important transcription factor that can promote the transcription of the DRD1 gene [51]. Previous studies have demonstrated that the endocrine disruptor bis(2-ethylhexyl) phthalate (DEHP) causes a decrease in cAMP and CREB levels in the brains of mice, which in turn results in neurotoxicity [52]. Consistent with this, in this study, the expression level of the CREB gene in zebrafish larvae was significantly decreased after being exposed to 3-isopropylphenol. This indicated that 3-isopropylphenol interfered with the normal function of the cAMP/PKA-CREB signaling pathway. CREB plays a crucial role in regulating the cell cycle and the expression of genes associated with cell differentiation. During the differentiation of neural stem cells, a decline in CREB expression may disrupt the normal differentiation of neurons and glial cells [53] and may heighten cellular sensitivity to apoptotic signals, rendering them more prone to apoptosis when subjected to external stimuli [54]. *Bad* is a pro-apoptotic protein that plays an important role in the cell apoptosis signaling pathway. *Bcl-2* is an anti-apoptotic protein that mainly functions by inhibiting the mitochondrial apoptosis pathway [55]. Zebrafish larvae experiments in this study showed that the expression of the *bad* gene was upregulated and the expression of the *bcl-2* gene was downregulated, indicating that exposure to 3-isopropylphenol led to excessive death of neuronal cells. Previous studies have shown that the neurotoxic mechanism of bis(2-ethylhexyl) phthalate (DEHP) in mice is caused by the synergistic effect of the cAMP/PKA/ERK1/2/CREB signaling pathway and the Ca^2+^ signaling pathway [52]. The above results indicate that exposure to 3-isopropylphenol can promote cell apoptosis in zebrafish larvae by inhibiting the cAMP/PKA signaling pathway and resulting in impaired neural development.

In this study, GNB1 and GNG2 were identified as two core targets. The molecular docking results confirmed their significant role in the neurotoxicity induced by 3-isopropylphenol, as they stably bound to the active site of 3-isopropylphenol with a binding energy lower than zero kcal/mol. Notably, these two core targets are key genes in the dopaminergic synaptic signaling pathway. As crucial components of the G protein heterotrimer, they are involved in the signaling process mediated by dopamine receptors. The experimental results showed a decrease in the gene expression of the dopamine receptor (*drd1*), indicating an inhibition of dopamine release. The reduced formation of βγ dimers by GNB1 and GNG2 led to a decrease in the efficiency of G protein-mediated signal transduction, thus suppressing the cascade conduction of the cAMP/PKA signaling pathway. Our research findings highlighted the importance of the neuroactive ligand–receptor interaction pathway, the dopaminergic synaptic signaling pathway, and the cAMP/PKA signaling pathway. These pathways are closely associated with nerve damage, suggesting that 3-isopropylphenol primarily induces neurotoxicity in zebrafish and promotes cell apoptosis, ultimately resulting in nerve damage and impaired motor behavior.

## 5. Conclusions

In summary, this study selected zebrafish as a model organism with the aim of predicting and verifying the potential toxic mechanisms and action targets of 3-isopropylphenol. This was achieved through the application of network toxicology, molecular docking, and RT-qPCR. The findings indicate that when zebrafish are exposed to 3-isopropylphenol, dopamine release is inhibited, the efficiency of signal transduction is reduced, and the cAMP/PKA signaling pathway is suppressed. Consequently, dopaminergic synaptic signal transmission is obstructed, leading to increased cell apoptosis, impaired neural development, and ultimately, defects in the motor behavior of larval zebrafish. This study utilized two important technical approaches, network toxicology and molecular docking, to investigate the neurotoxicity and mechanisms of 3-isopropylphenol from different angles. It also comprehensively analyzed the neurotoxic effects of 3-isopropylphenol on zebrafish larvae. This not only helps to accurately assess the potential harm of 3-isopropylphenol to aquatic organisms in the actual environment, filling part of the gaps in environmental risk assessment in this field, but also provides a new perspective and data support for a deeper understanding of the mechanisms by which pollutants interfere with the neural development of aquatic organisms, contributing to the further development of related fields.

## Data Availability

The raw data supporting the conclusions of this article will be made available by the authors upon request.

## Supplementary Materials

The following supporting information can be downloaded at: https://www.mdpi.com/article/10.3390/toxics13040274/s1. Table S1: The primer sequences for QPCR; Table S2: Candidate targets screened from the PPI network.
