# Supplementary material for: Mechanistic Insights into 3-Isopropylphenol-Induced Neurotoxicity in Zebrafish: A Network Toxicology and Molecular Docking Approach
Source: Toxics. 2025 Apr 3;13(4):274. doi: 10.3390/toxics13040274 (PMC12031193; doi:10.3390/toxics13040274)
Supplement: Supplementary file 1 [file toxics-13-00274-s001.zip › toxics-3541709-supplementary.pdf]

Supplementary materials

Supplementary Table 1
The primer sequences for QPCR.

Gene	Forward (5'-3')	Reverse (5'-3')
β-actin	TCTGGCATCACACCTTCTACAAT	TGTTGGCTTTGGGATTCAGG
elavl3	AGACAAGATCACAGGCCAGAGCTT	TGGTCTGCAGTTTGAGACCGTTGA
syn2a	GTGACCATGCCAGCATTTC	TGGTTCTCCACTTTCACCTT
gfap	GGATGCAGCCAATCGTAAT	TTCCAGGTCACAGGTCAG
gap43	TGCTGCATCAGAAGAACTAA	CCTCCGGTTTGATTCCATC
drd1	ACGCTGTCCATCCTTATCTC	TGTCCGATTAAGGCTGGAG
drd2a	TGGTACTCCGGAAAAGACG	ATCGGGATGGGTGCATTTC
cAMP	AGGATAACAAGAGATTTGCCC	CCAATCTTCTCTTTAGATTTCCGG
PKA	GCGAGATTTCAAGATCCAGACTC	ATAAGACAGAAGGCCGGCAG
CREB	AGTTGTTGTTCAAGCTGCCTCTG	TCCTTAAGTGCTTTTAGCTCCTCA
gnb1b	TATACCACAAATAAGGTCCACG	GTAATTCCCTGAAGGAGCG
gng2	AACAACACAGCTAGCATCG	TGCTTTGGAGACCTTTATTCTG
bad	CCACAACGAGGACTACACC	CATGCTCGAGTCACTCTGCGGGGCGCG
bcl-2	AGGAAAATGGAGGTTGGGATG	TGTTAGGTATGAAAACGGGTGGA

Supplementary Table 2
Candidate targets screened from the PPI network.

Name	Degree	Closeness centrality	Betweenness centrality	Topological coefficient
GNB1	16	0.288135593220339	0.2156268568033274	0.2743055555555556
GNG2	15	0.2857142857142857	0.18383838383838383	0.2851851851851852
GNAI1	11	0.2764227642276423	0.11051693404634579	0.3333333333333333
NCOR2	10	0.23129251700680273	0.245395127748069	0.3
ESR1	7	0.265625	0.5433749257278668	0.30952380952380953
DRD2	6	0.3035714285714286	0.5294711824123589	0.4803921568627451
HTR2A	5	0.26356589147286824	0.022103386809269158	0.5
DRD3	5	0.23287671232876714	0.013606654783125371	0.575
HDAC1	5	0.2236842105263158	0.016339869281045742	0.45
HTR2C	4	0.22972972972972974	0	0.625
HTR2B	4	0.22972972972972974	0	0.625
MTNR1A	4	0.22972972972972974	5.941770647653E-4	0.703125
HDAC2	4	0.2222222222222222	0.0148544266191325	0.5208333333333334
CXCR2	4	0.22972972972972974	0	0.71875
CCR2	4	0.22972972972972974	0	0.71875
HDAC3	3	0.19101123595505617	0	0.6333333333333333

LTB4R	3	0.22818791946308722	0	0.875
MTNR1B	3	0.22818791946308722	0	0.6458333333333334
OPRD1	3	0.22818791946308722	0	0.875
DRD1	3	0.22972972972972974	0	0.7058823529411765
CNR2	3	0.22818791946308722	0	0.875
AR	3	0.2207792207792208	0	0.6111111111111112
JAK2	2	0.18085106382978725	0.058823529411764705	0.5
HTR6	2	0.22666666666666666	0	0.96875
IGF1R	2	0.21656050955414013	0.1140819964349376	0.5
ESR2	2	0.21935483870967742	0	0.7083333333333334
SLC6A3	2	0.30088495575221236	0.5133689839572192	0.5
DRD4	2	0.23943661971830985	0.00802139037433155	0.75
SNCA	2	0.2931034482758621	0.5080213903743315	0.5
CALM1	2	0.2809917355371901	0.49910873440285203	0.5
JAK1	1	0.15384615384615385	0	0
HDAC8	1	0.18888888888888888	0	0
HDAC4	1	0.18888888888888888	0	0
HDAC10	1	0.18888888888888888	0	0
ESRRG	1	0.18888888888888888	0	0
